# miRNA-126a plays important role in myoblast and endothelial cell interaction

**DOI:** 10.1038/s41598-023-41626-z

**Published:** 2023-09-12

**Authors:** Bartosz Mierzejewski, Maria Anna Ciemerych, Wladyslawa Streminska, Katarzyna Janczyk-Ilach, Edyta Brzoska

**Affiliations:** Department of Cytology, Faculty of Biology, University of Warsaw, Miecznikowa 1 St, 02-096 Warszawa, Poland

**Keywords:** Cell biology, Stem cells

## Abstract

Muscle satellite cells (SCs) are stem cells and the main players in skeletal muscle reconstruction. Since satellite cells are located near or in direct contact with blood vessels their niche is formed, *inter alia,* by endothelial cells. The cross-talk between satellite cells and endothelial cells determines quiescence or proliferation of these cells. However, little is known about the role of miRNA in these interactions. In the present study we identified miRNA that were up-regulated in SC-derived myoblasts treated with stromal derived factor-1 (SDF-1) and/or down-regulated in cells in which the expression of CXCR4 or CXCR7, that is, SDF-1 receptors, was silenced. SDF-1 is one of the important regulators of cell migration, mobilization, skeletal muscle regeneration, and angiogenesis. We hypothesized that selected miRNAs affect SC-derived myoblast fate and interactions with endothelial cells. We showed that miR-126a-3p inhibited both, myoblast migration and fusion. Moreover, the levels of *Cxcl12,* encoding SDF-1 and *Ackr3,* encoding CXCR7, were reduced by miR-126a-3p mimic. Interestingly, the miR-126a-3p mimic significantly decreased the level of numerous factors involved in myogenesis and the miR-126a-5p mimic increased the level of *Vefga.* Importantly, the treatment of endothelial cells with medium conditioned by miR-126-5p mimic transfected SC-derived myoblasts promoted tubulogenesis.

## Introduction

Regeneration of skeletal muscles is a complex process with the essential role of satellite cells (SCs), i.e., unipotent stem cells connected with myofibers. In response to skeletal muscle damage, SCs are activated, start to proliferate, differentiate into myoblasts and then myocytes that fuse to form multinucleated myotubes and muscle fibers. This process is regulated by myogenic regulatory factors (MRFs), such as MYOD, MYF5, and myogenin. However, extracellular factors also play an important supervisory role and regulate SC quiescence, self-renewal, proliferation, and differentiation during skeletal muscle reconstruction. Factors, such as cytokines and growth factors, are released by damaged myofibres, inflammatory cells, fibroblasts, adipogenic/fibrogenic precursors (FAPs), and endothelial cells^1^. In addition, the role of many factors such as WNT, NOTCH, or sphingolipid signaling, extracellular matrix, hepatocyte growth factor (HGF), fibroblast growth factor (FGF), insulin-like growth factor (IGF), or stromal derived factor -1 (SDF-1) was previously described^2^. Furthermore, SCs influence the niche through cell–cell interaction and autocrine or paracrine signals and SC niche is reorganized during the skeletal muscle reconstruction^2^. Among important players during the formation of the SC niche are endothelial cells. In intact adult muscles most SCs are located close to the capillaries^3^. As a result, in case of the injury, endothelial cells could promote SC-derived myoblast proliferation in IGF, HGF, FGF, platelet-derived growth factor-BB (PDGF-BB), and vascular endothelial growth factor (VEGF) dependent manner^3^. On the other hand, angiopoietin-1 (ANGPT-1) secreted by endothelial cells promote SC quiescence^3^. In addition, endothelial cells, SCs, and myoblasts synthesize VEGF that regulates both angiogenesis and myogenesis^4^. However, little is known about the role of microRNA (miRNA) in these interactions and SC niche homeostasis.

The miRNAs control gene expression by silencing specific mRNAs or by suppression of protein translation. The group of miRNAs particularly important during skeletal myogenesis is called myomiRs and includes miR-1, miR-133a, miR-133b, miR-486, miR-499, miR-206, miR-208a, miR-208b, and miR-206^5^. In the present study we focus on miR-126-3p, miR-126-5p miR-324-3p, and miR-339-5p that we showed to be regulated by SDF-1 and its receptors, that is, CXCR4 and CXCR7. SDF-1 is one of the important regulators of cell migration, mobilization, skeletal muscle regeneration, and angiogenesis^6–10^.

As mentioned above, miR-126 is among the microRNAs implicated in angiogenesis and vascular integrity^11, 12^. In endothelial cells it inhibits the SDF-1 expression and its silencing elevates SDF-1 levels^13, 14^. Furthermore, in endothelial cells cultured in vitro under the hypoxic conditions miR-126 level decreases^15^. In the absence of miR-126 neovascularization associated with myocardial infraction or retinal vascular development and remodeling is distrupted^11, 12^. The miR-126 is encoded in chromosome 2 (mouse) or 9 (human) within the epidermal growth factor like domain multiple 7 *(EGFL7)* gene intron. *EGFL7* gene encodes endothelial cell protein that contains two epidermal growth factor-like domains. In response to VEGF or FGF, miR-126 targets negative regulators participating in MAPK and PI3K signaling, including Sprouty-related protein 1 (SPRED-1) and phosphoinositol-3 kinase regulatory subunit 2 (PIK2R2/p38-β)^12^. In vitro knockdown of miR-126 decreased human umbilical vein endothelial cells (HUVEC) proliferation, migration, and tubulogenesis, i.e., in vitro vascular tube formation in Matrigel^12^. Furthermore, miR-126 was shown to suppress inflammation by targeting VCAM-1 and s1PR2^12^. Also in vivo studies showed the pro-angiogenic role of miRNA-126^12^.

miR-324-3p is a molecule that is involved in cancer development and progression. It was described as an important inducer of colon cancer development and breast cancer invasion and metastasis. It inhibits the expression of CUE domain containing 2 (CUED2), a negative regulator of NFκB signaling. The low level of CUED2 promotes the production of inflammatory cytokines by macrophages and NFκB activity which was shown to be crucial for breast cancer EMT and metastasis^16, 17^. miR-324-3p was also shown to suppress migration and invasion of nasopharynegral carcinoma by targeting WNT2B and hepatocellular carcinoma cells by directly inhibiting specificity protein 1 (SP1) and E26 transformation specific 1 (ETS1)^18^. Both SP1 and E26 can modulate the expression and activity of matrix metalloproteinases MMP2 and MMP9, the main regulators of extracellular matrix (ECM) remodeling^19^. Furthermore, miR-324-3p inhibited the differentiation of C2C12 mouse myoblasts^20^.

miR-339-5p has been described as a tumor suppressor of many types of cancer. Its up-regulation inhibited migration and invasion of breast cancer cells, ovarian cancer cells, and lung adenocarcinoma by targeting B cell lymphoma-6 (BCL6)^21^. miR-324-3p was also shown to inhibit smooth muscle cell proliferation by targeting FGF signaling and regulate bone marrow-derived mesenchymal stem cells (BMSC) osteogenic differentiation via targeting distal-less homeobox 5 (DLX5), a transcription factor involved in bone development^22^.

In the present study, we hypothesized that selected miRNAs impact cell proliferation, migration, and/or differentiation. Two populations of cells present in the skeletal muscle niche were analyzed, i.e., satellite cell-derived myoblasts and endothelial cells.

## Results

### Changes in miRNA level after SDF-1 treatment or Cxcr4 or Cxcr7 down-regulation

**Figure 1:**
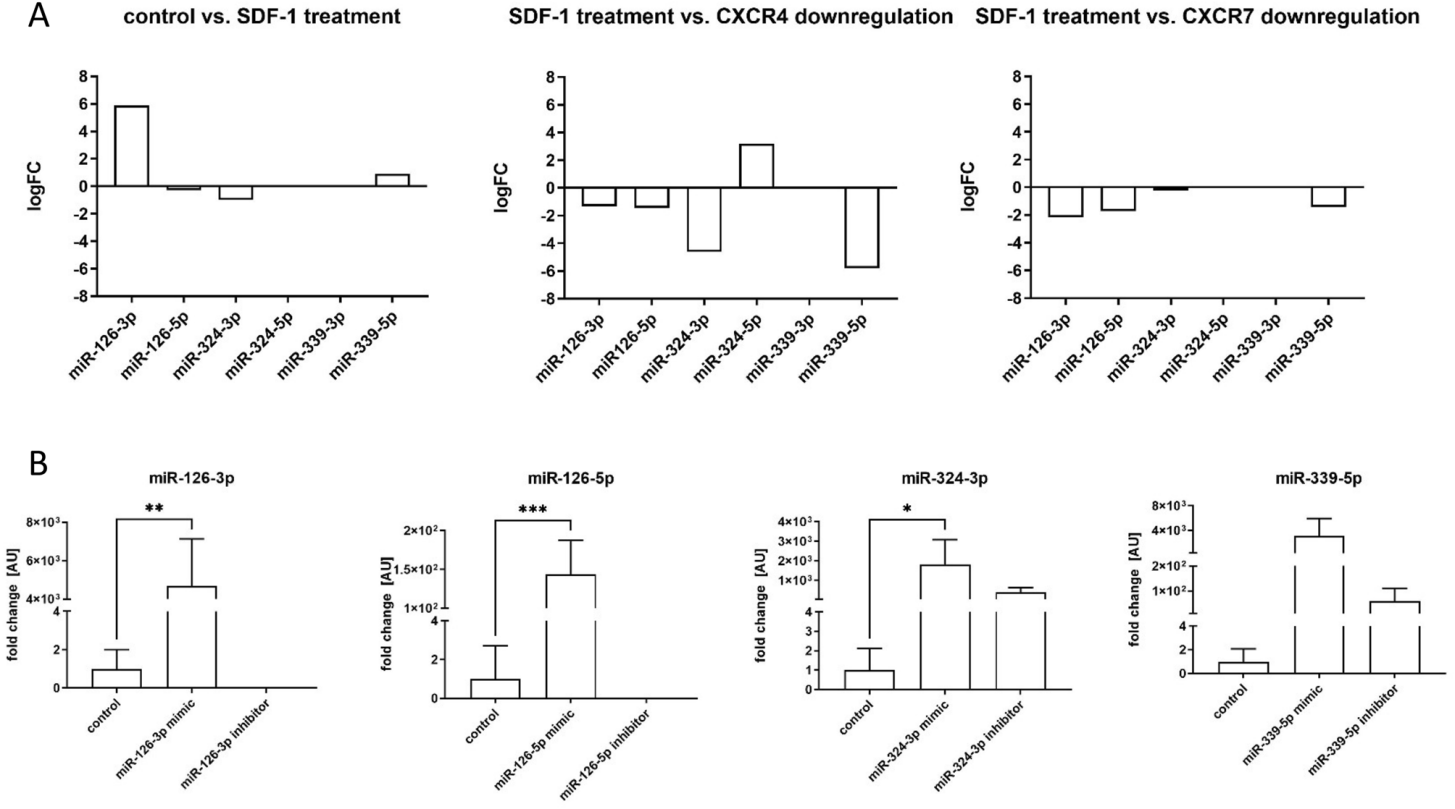
Analyzes of miRNA level and transfection efficiency of mouse SC-derived myoblasts; (**A**) The changes of the levels of miR-126-3p, miR-126-5p, miR-324-3p, miR-324-5p, miR-339-3p, and miR-339-5p in SC-derived myoblasts after SDF-1 treatment, down-regulation of CXCR4 or down-regulation of CXCR7 assessed by next generation sequencing; (**B**) Expression of miR-126-3p, miR-126-5p, miR-324-3p, and miR-339-5p in control SC-derived myoblasts or SC-derived myoblasts transfected with mimic or inhibitors. Differences were considered statistically significant when *p <* 0.05 (marked with asterisks, *p < 0.05; **p < 0.005; ***p < 0.001).

In our previous study, we noticed that SDF-1 impacts SC-derived myoblasts migration and participation in skeletal muscles regeneration^6, 8–10, 23^. However, little is known about the impact of miRNA on SDF-1 signaling. To select miRNAs engaged in SDF-1 regulation the next generation sequencing (NGS) was performed using control, SDF-1 treated, SDF-1 treated and *Cxcr4* siRNA or SDF-1 treated and *Cxcr7* siRNA transfected SC-derived myoblasts. NGS allowed us to analyze the level of microRNA which expression changes in control and treated cells. We selected 6 molecules: miR-126a-3p, miR-126a-5p, miR-324-3p, miR-324-3p-5p, miR-339-5p-3p, miR-339-5p-5p that level differed significantly between control SC-derived myoblasts, those treated with SDF-1, and those in which *Cxcr4* or *Cxcr7* were down-regulated (Fig. 1A). The level of miR-126a-3p and miR-339-5p-5p increased in SDF-1 treated SC-derived myoblasts, compared to control ones. On the other hand, the level of miR-126a-3p, miR-126-5p, miR-324-3p, and miR-339-5p-5p decreased in SC-derived myoblasts in which *Cxcr4* or *Cxcr7* were downregulated, if compared to those treated with SDF-1. Thus, SC-derived myoblasts were transfected with miRNA126-3p, miR-126-5p, miR-324-3p, miR-339-5p mimics or selected microRNA inhibitors to follow changes in cell proliferation, migration, differentiation, and transcriptome. First, we confirmed that transfection with specific microRNA mimics or inhibitors, respectively, led to up-regulation or downregulation of specific miRNAs in SC-derived myoblasts (Fig. 1B).

### The influence of miRNA mimics and inhibitors transfection on SC-derived myoblasts proliferation, migration, and fusion

**Figure 2:**
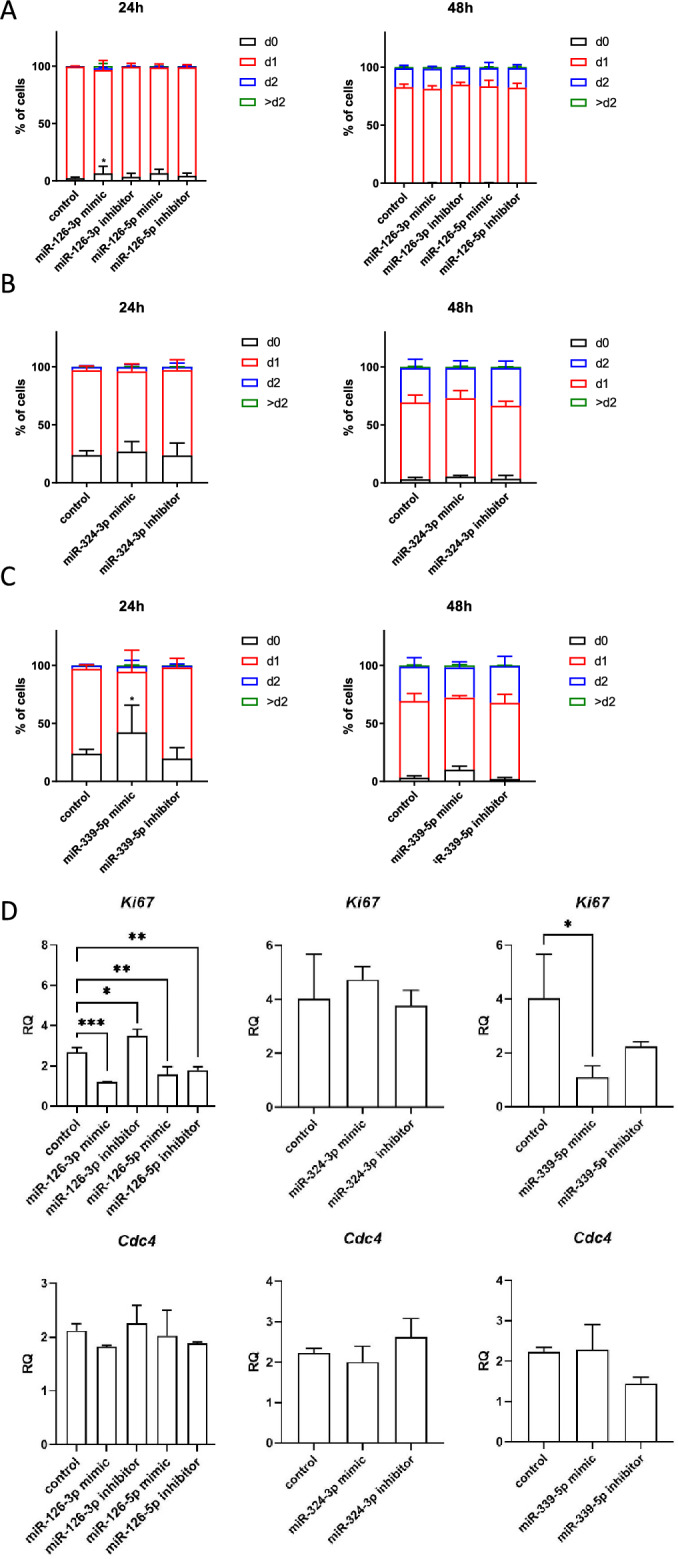
Impact of miR-126-3p, miR-126-5p, miR-324-3p, and miR-339-5p on mouse SC-derived myoblast proliferation. CFSE assay of control SC-derived myoblasts or SC-derived myoblasts transfected with miRNA mimics or inhibitors 24 h or 48 h after adding CFSE; (**A**) SC-derived control myoblasts and SC-derived myoblasts transfected with miR-126-3p or miR-126-5p mimics or their inhibitors; (**B**) SC-derived control myoblasts and SC-derived myoblasts transfected with mimic or inhibitor of miR-324-3p; (**C**) SC-derived control myoblasts and SC-derived myoblasts transfected with mimic or inhibitor of miR-339-5p. The proportion of cells that underwent one (d1), two (d2) or more (> d2) divisions were shown; (**D**) the level of *Ki67* and *Cdc4* transcripts in control SC-derived myoblasts and SC-derived myoblasts transfected with miR-126-3p, miR-126-5p, miR-324-3p, and miR-339-5p mimics or their inhibitors. Differences were considered statistically significant when *p <* 0.05 (marked with asterisks, *p < 0.05).

The proliferation of SC-derived myoblasts transfected with either miR-126-3p, or miR-126-5p, or miR-324-3p, or miR-339-5p mimics or microRNA inhibitors was analyzed using the flow cytometry of cells cultured in the presence of CFSE for 24 h or 48 h (Fig. 2A–C). No significant changes in proliferation of SC-derived myoblasts transfected with mimics or inhibitors, as compared to control, were observed at selected time points. The only exception were the cells transfected with miR126-3p or miR339-5p mimics. In this case we observed decreased proliferation at 24 h (Fig. 2A–C). In these cases, the number non-divided cells increased. Proliferation of SC-derived myoblasts was also assessed by the analysis of the expression of *Ki67* and *Cdc4* (encoding cell division control protein 4), i.e., the markers of dividing cells (Fig. 2D). The transfection of SC-derived myoblasts with either miR-126-3p, or miR-126-5p, or miR-339-5p mimics or miR-126a-5p inhibitor significantly decreased the *Ki67* mRNA level. Furthermore, transfection with the miR-126a-3p inhibitor increased *Ki67* mRNA level. However, the level of *Cdc4* mRNA did not differ, as compared to control cells. Altogether, this analyses suggest that miR126-3p and miR-339-5p could impact SC-derived myoblast proliferation.

**Figure 3:**
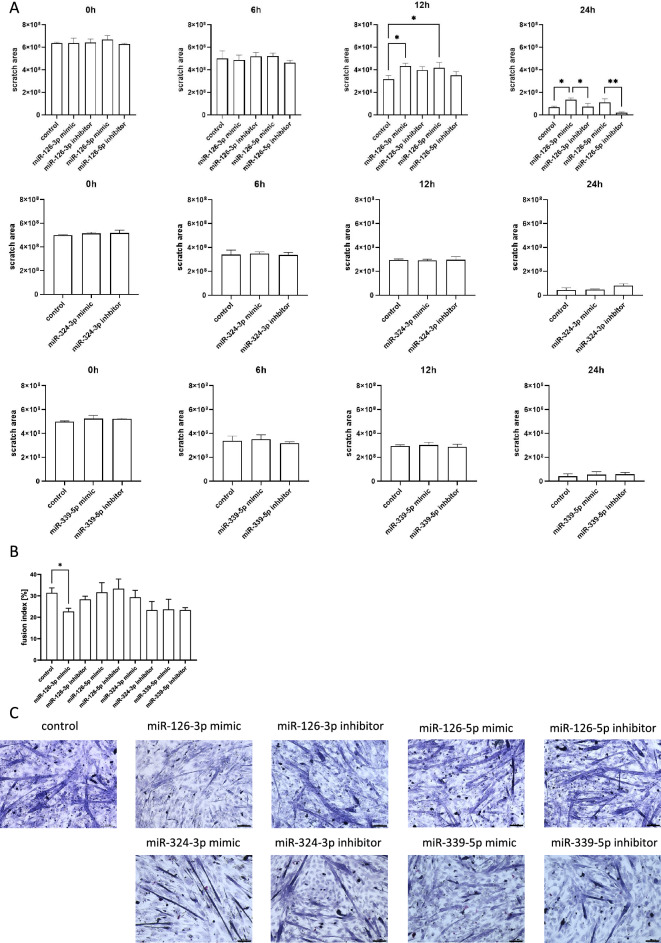
Impact of miR-126-3p, miR-126-5p, miR-324-3p, and miR-339-5p on mouse SC-derived myoblast migration and fusion. (**A**) Migration of control or transfected SC-derived myoblasts analyzed 6, 12, and 24 h after performing a scratch; (**B**) Fusion index of control or transfected SC-derived myoblasts. Differences were considered statistically significant when *p <* 0.05 (marked with asterisks, *p < 0.05; **p < 0.005). (**C**) Representative images of control or transfected SC-derived myoblasts fusion.

Then, the migration capacity of SC-derived myoblasts transfected with either miRNA126-3p, or miR-126-5p, or miR-324-3p, or miR-339-5p mimics or microRNA inhibitors was assessed using scratch assay (Fig. 3A). Migration was analyzed after 6, 12, and 24 h after the scratch was made. MiR-126-3p or miR-126-5p mimic transfected SC-derived myoblast migration was less effective than that of control cells (Fig. 3A). Furthermore, cells transfected with either miR-126-3p or miR-126-5p inhibitor migrated more effectively than those transfected with microRNA mimics. Changes in migration of SC-derived myoblast transfected with either miR-324-3p, or miR-339-5p mimics or inhibitors were not observed (Fig. 3A). To follow changes in SC-derived myoblast differentiation, the fusion index was analyzed (Fig. 3B,C). The SC-derived myoblasts were cultured in the presence of medium that supports myogenic differentiation for 5 days. The fusion index of SC-derived myoblast transfected with miR-126a-3p mimic was significantly lower than that of control cells (Fig. 3B,C). The fusion index of cells transfected either with miR-324-3p, or miR-339-5p mimics or their inhibitors did not differ, as compared to control ones (Fig. 3B,C).

### The influence of miRNA mimics and inhibitors transfection on selected transcripts

**Figure 4:**
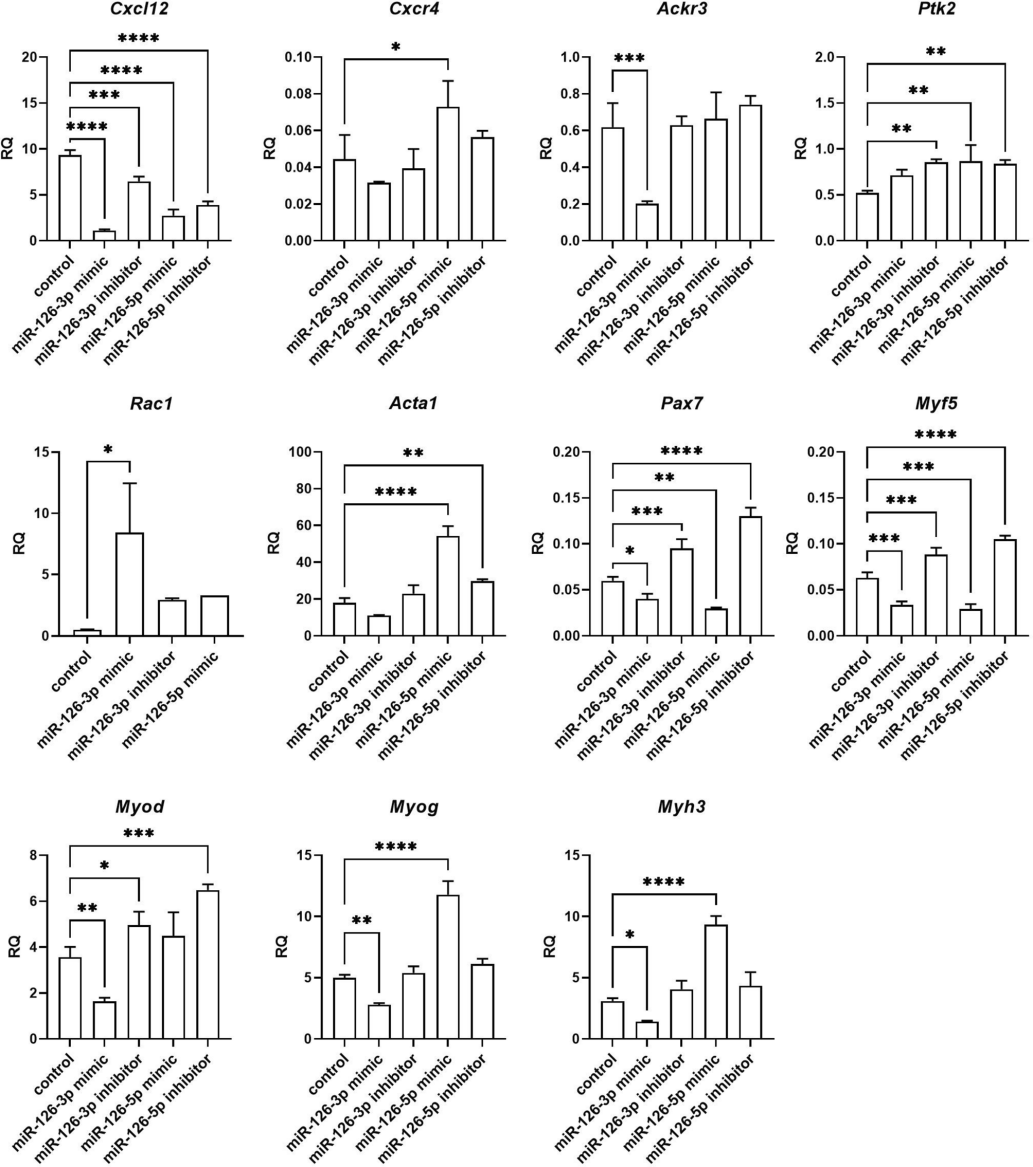
Relative expression of selected transcripts of control mouse SC-derived myoblasts or SC-derived myoblasts transfected with miR-126-3p or miR-126-5p mimics or their inhibitors. Differences were considered statistically significant when *p <* 0.05 (marked with asterisks, *p < 0.05; **p < 0.005; ***p < 0.001; ****p < 0.0001).

**Figure 5:**
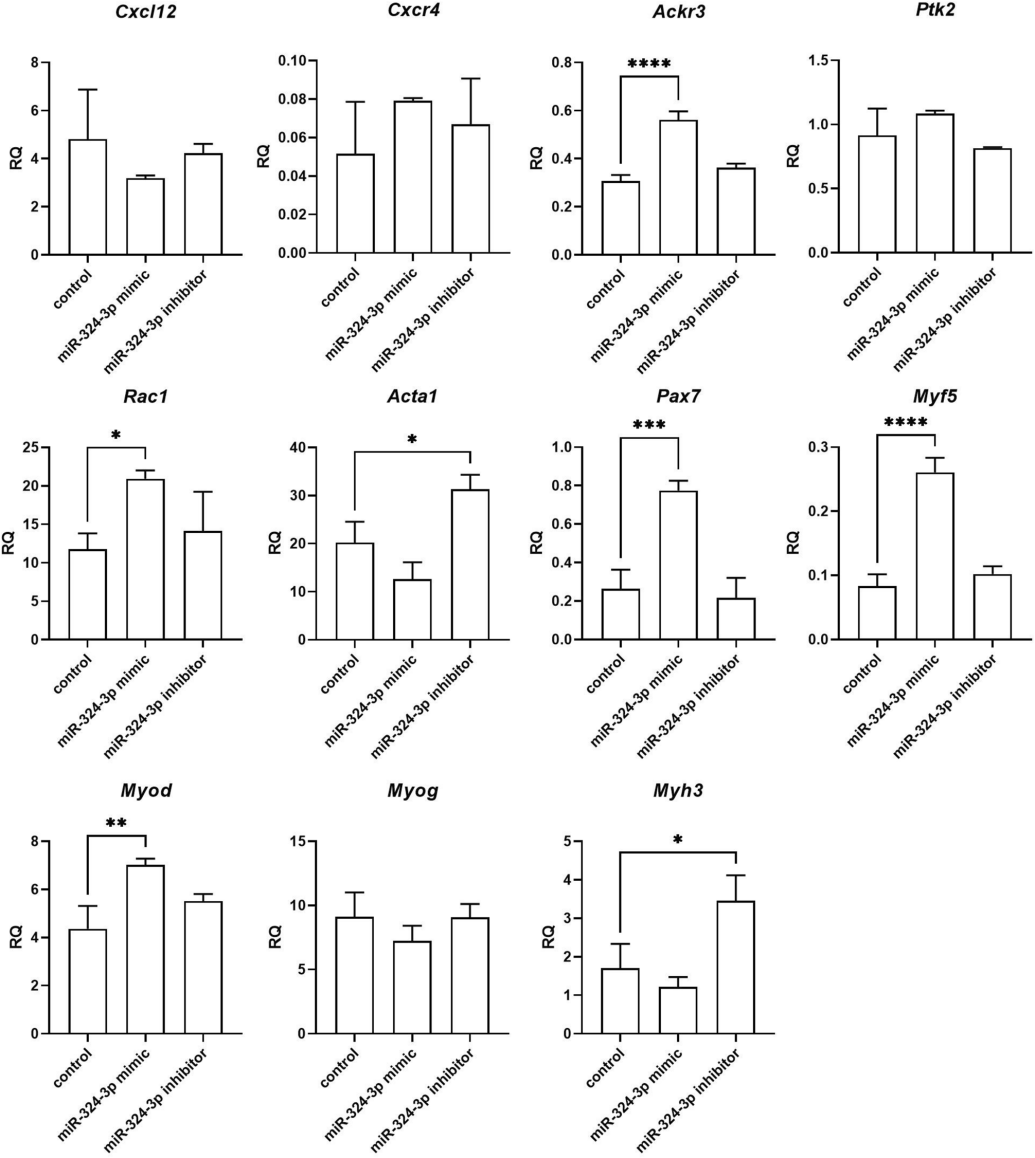
Relative expression of selected transcripts of control mouse SC-derived myoblasts and SC-derived myoblasts transfected with miR-324-3p mimic or its inhibitor. Differences were considered statistically significant when *p <* 0.05 (marked with asterisks, *p < 0.05; **p < 0.005; ***p < 0.001; ****p < 0.0001).

**Figure 6:**
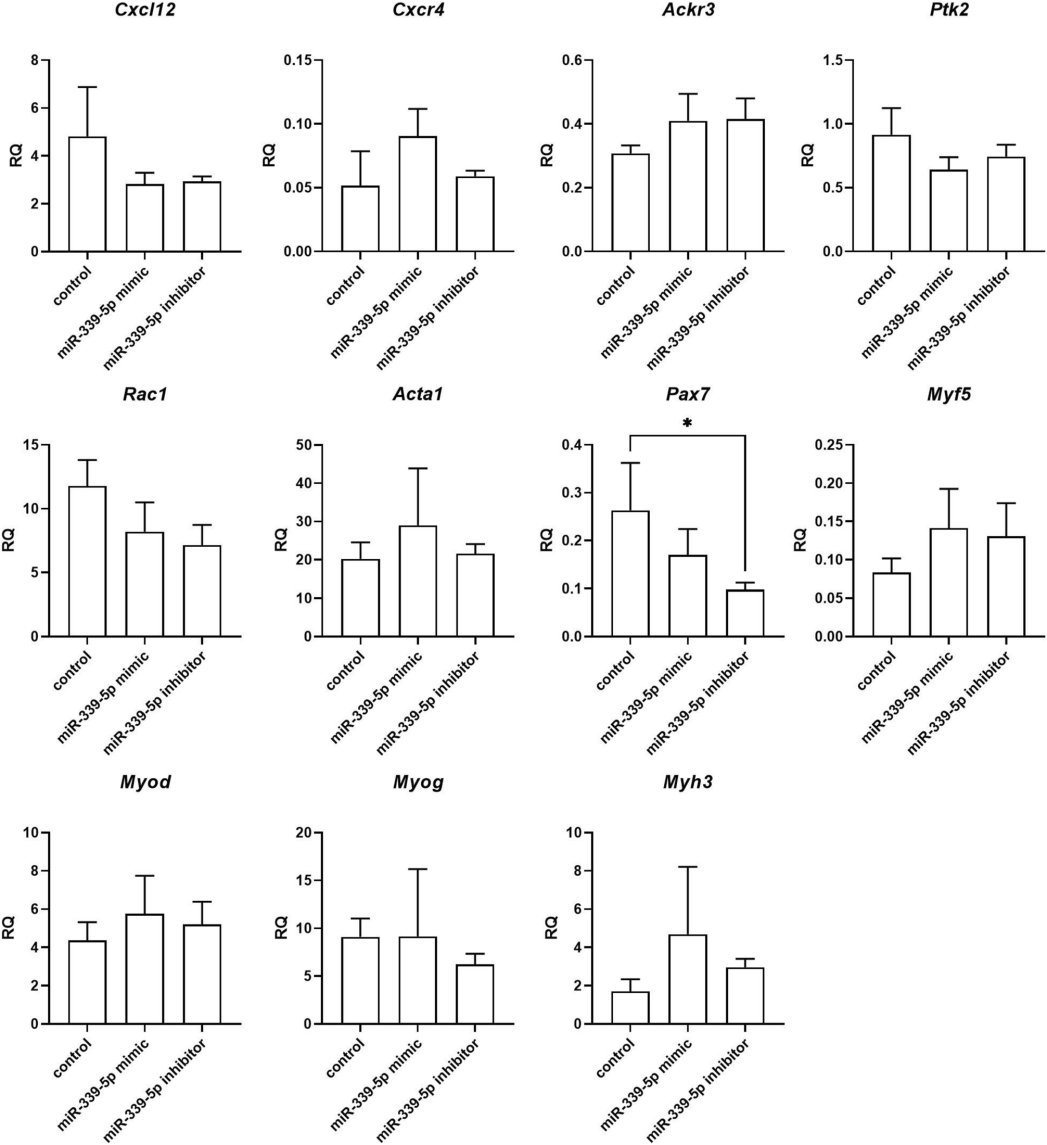
Relative expression of selected transcripts of control mouse SC-derived myoblasts and SC-derived myoblasts transfected with miR-339-5p mimic or inhibitor. Differences were considered statistically significant when *p <* 0.05 (marked with asterisks, *p < 0.05; **p < 0.005; ***p < 0.001; ****p < 0.0001).

**Figure 7:**
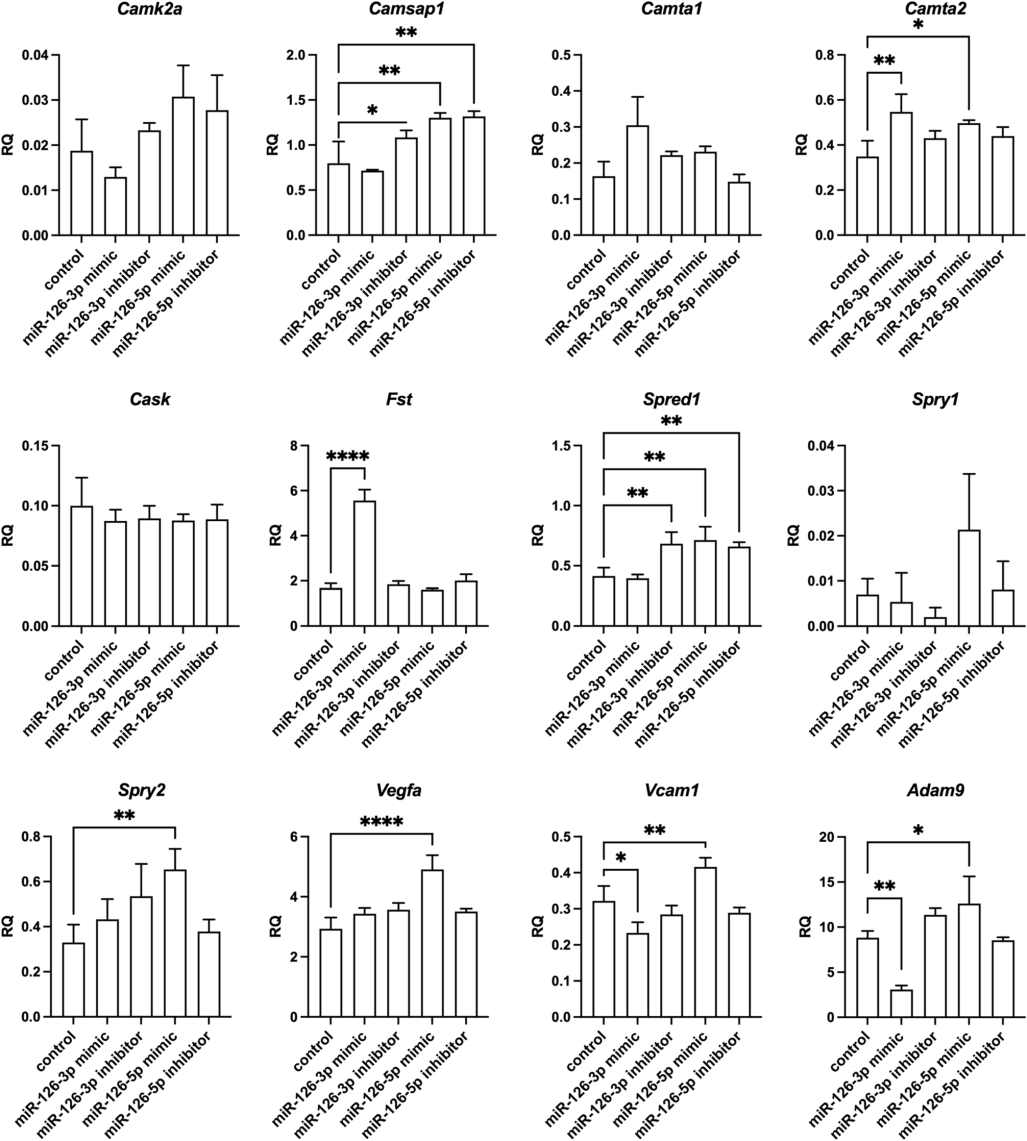
Relative expression of transcripts predicted by databases to be regulated by miR-126-3p or miR-126-5p of control mouse SC-derived myoblasts and SC-derived myoblasts transfected with miR-126-3p or miR-126-5p mimics or their inhibitors. Differences were considered statistically significant when *p <* 0.05 (marked with asterisks, *p < 0.05; **p < 0.005; ***p < 0.001; ****p < 0.0001).

Changes in selected transcript levels were analyzed in SC-derived myoblasts transfected with either miRNA126-3p, or miR-126-5p, or miR-324-3p, or miR-339-5p mimics or inhibitors (Figs. 4, 5, 6, 7). The level of transcripts encoding proteins involved in SDF-1 signaling, cell proliferation, migration, and myogenic differentiation was examined. First, we focused on transcripts involved in SDF-1 signaling, such as *Cxcl12*, *Cxcr4*, and *Ackr3* encoding SDF-1, CXCR4, and CXCR7, respectively. A significantly lower level of *Cxcl12* and *Ackr3* was observed in SC-derived myoblasts transfected with miR-126a-3p mimic (Fig. 4). A significantly lower level of *Cxcl12* and the higher level of *Cxcr4* was observed in SC-derived myoblast transfected with miR-126a-5p mimic (Fig. 4). Transfection with either miR-324-3p, or miR-339-5p mimics, or inhibitors did not change the level of transcripts encoding proteins involved in SDF-1 signaling, except for miR-324-3p mimic that increased the level of *Ackr3* (Fig. 5). Changes in the level of transcripts encoding factors involved in cell proliferation were observed (Figs. 4, 5, 6). Then we analyzed the level of transcripts encoding factors involved in cytoskeleton reorganization and cell migration, such as *Ptk2* and *Rac1*, which encode focal adhesion kinase (FAK) and Ras-related C3 botulinum toxin substrate 1 (RAC1), respectively (Figs. 4, 5, 6). *Ptk2* expression increased significantly in SC-derived myoblasts transfected with either miR-126a-3p inhibitor, or miR-126a-5p mimic, or miR-126a-5p inhibitor. The level of *Rac1* increased significantly in miR-126a-3p or miR-324-3p mimic transfected SC-derived myoblasts. Then, the levels of transcript encoding proteins engaged in myogenic differentiation, such as transcription factors: *Pax7, Myod, Myf5, Myog*, and structural protein transcripts of muscle cells, such as *Acta1, Myh3* were analyzed (Figs. 4, 5, 6)*.* Interestingly, miR-126a-3p mimic transfection significantly decreased the level of *Pax7, Myod, Myf5, Myog,* and *Myh3* in SC-derived myoblasts*.* Furthermore, miR-126-5p mimic transfection significantly decreased the level of *Pax7, Myf5* and increased the level of *Myog, Myh3,* and *Acta1.* The effect was reversed when miR-126a inhibitors were used. Furthermore, miR-324-3p mimic transfection significantly increased the level of *Pax7, Myod, Myf5,* and the miR-126-5p inhibitor increased the level of *Acta1* and *Myh3.* In summary, miR-126-3p mimic transfection impacted the level of transcripts encoding factors involved in SDF-1 signaling, proliferation, migration, and myogenic differentiation and corresponds to changes observed during proliferation, migration, and fusion. The effect of miR-324-3p was observed mainly in case of myogenic factor transcript levels. However, these changes did not translate into differences in the fusion index. Thus, in further experiments we focused on the miR-126 function.

### The miR-126 targets

According to miR Data Base, Target Scan Mouse data base, mirWalk2, mirTarbase, among the predicted *miR-126* targets are transcripts encoding adhesion proteins: *Adam9* (a disintegrin and metallopeptidase domain 9), *Vcam1*; cytokine and growth factors: *Vegfa*, *Cxcl12, Cxcr4, Fst* (follistatin), *Spred1* (sprouty protein with EVH-1 domain 1), *Spry1* and *Spry2* (Sprouty RTK signaling antagonist 1), calcium/calmodulin dependent proteins: *Camk2a* (calcium/calmodulin dependent protein kinase II alpha), *Camsap1* (calmodulin regulated spectrin-associated protein 1), *Camta1* and *Camta2* (calmodulin-binding transcription activator), *Cask* serine protein kinase). As described above, we confirmed the down-regulation of *Cxcl12* in miR-126-3p or miR-126-5p mimic transfected SC-derived myoblasts. Then we focused on other miR-126 targets and analyzed its level in transfected SC-derived myoblasts (Fig. 7). We did not observe down-regulation of *Camk2a, Camsap1, Camta1, Camta2, Cask, Fst, Spred1, Spry1, or Spry2* . However, the level of *Camsap1* and *Spred1* increased in miR-126-3p inhibitor transfected SC-derived myoblasts. Furthermore, the level of *Camta1*, *Camta2*, *Fst* increased in miR-126-3p mimic transfected SC-derived myoblasts. However, the level of *Adam9* and *Vcam1* decreased significantly in miRNA-126a-3p mimic transfected SC-derived myoblasts. Importantly, *Adam9*, *Vcam1,* and *Vegfa* levels increased significantly in miRNA-126a-5p mimic transfected SC-derived myoblasts.

### The impact of miR-126 transfected SC-derived myoblasts conditioned medium on endothelial cells tubulogenesis

**Figure 8:**
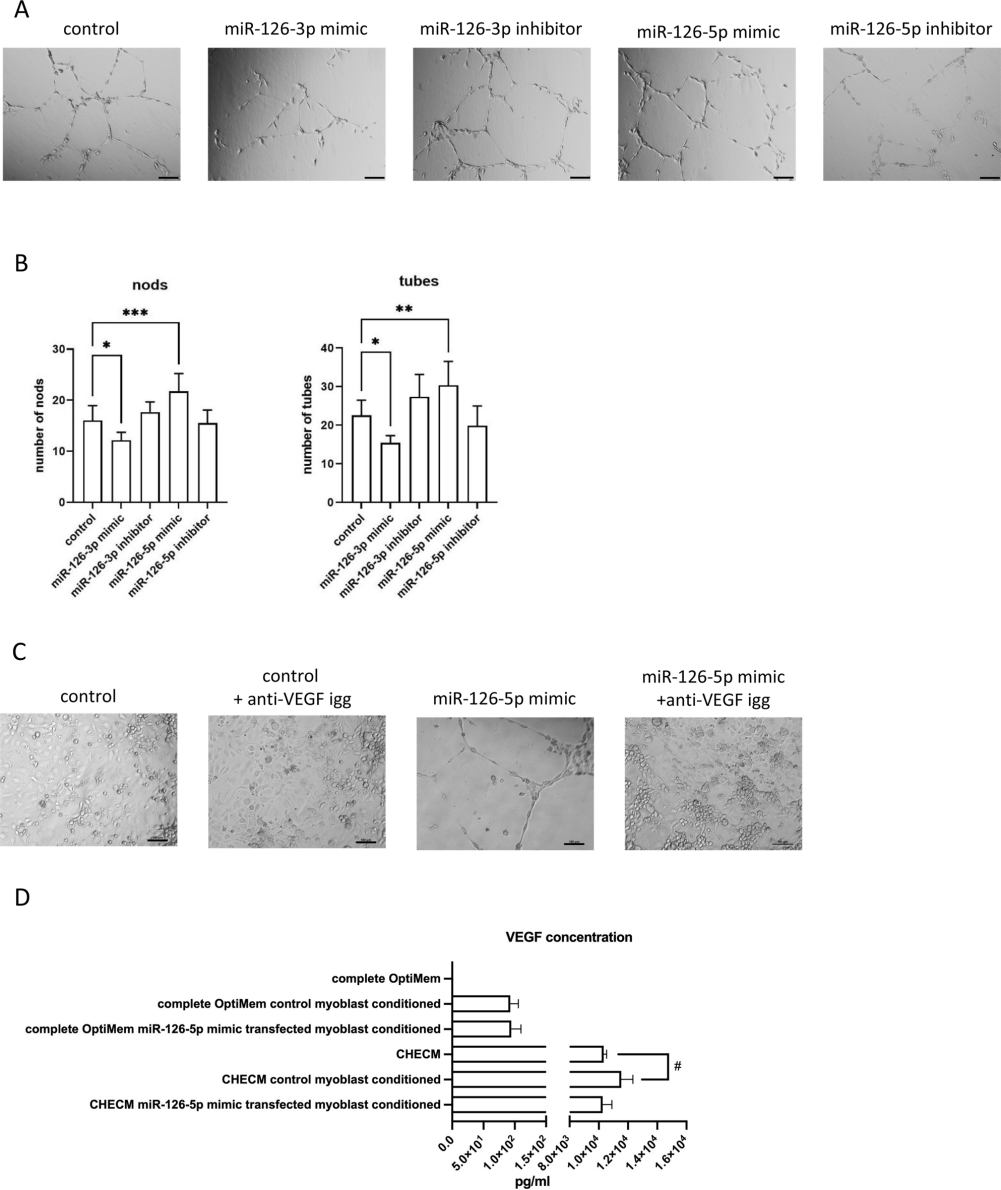
Impact of medium conditioned by the mouse control SC-derived myoblast or SC-derived myoblast transfected with miR-126-3p and miR-126-5p mimics or their inhibitors on PMECs. (**A**) Representative images of tube formation by PMECs cultured in control medium or medium collected from control or transfected SC-derived myoblasts (18 h); (**B**) Number of branching points (nods) and number of tubes formed by PMECs cultured in control medium or medium collected from control or transfected SC-derived myoblasts (18 h). Differences were considered statistically significant when *p <* 0.05 (marked with asterisks, *p < 0.05; **p < 0.005; ***p < 0.001). Scale: 100 µm. (**C**) The impact of anti-VEGF antibody present in culture medium or medium conditioned by the mouse control SC-derived myoblast or SC-derived myoblast transfected with miR-126-5p mimics on PMECs tubulogenesis (16 h). (**D**) The level of VEGF protein in complete OptiMem or Complete Human Endothelial Cell Medium (CHECM), or complete OptiMem collected from above control or transfected SC-derived myoblasts (48 h after transfection) or CHECM conditioned with control or transfected SC-derived myoblasts presence for 24 h. Differences were considered statistically significant when *p <* 0.05 (marked with asterisks, *p < 0.05; **p < 0.005; ***p < 0.001, when compared to OptiMem or marked with hashtag, #*p <* 0.05; ##*p <* 0.005; ###*p <* 0.001, when compared to CHECM).

In the present study, we showed that myoblast transfection with miR-126-5p, which expression significantly increased after SDF-1 treatment, increased *Vefga* level (Fig. 7). To assess the functional impact of miR-126-5p treated SC-derived myoblasts on endothelial cells we used primary human skeletal muscle microvascular endothelial cells (PMECs). Importantly, treatment of PMECs with medium conditioned by miR-126-5p transfected SC-derived myoblasts promoted tubulogenesis (Fig. 8A,B). On the other hand, the presence of medium conditioned by miR-126-3p transfected SC-derived myoblast inhibited endothelial cell differentiation (Fig. 8A,B). Next, the PMECs were cultured in miR126-5p transfected myoblast conditioned medium in presence of anti-VEGF antibody (Fig. 8C). PMEC tubules were formed in miR126-5p transfected myoblast conditioned medium already after 16 h. At the same time control myoblast conditioned medium did not promote tubule formation. Moreover, PMEC tubulogenesis was impaired in the presence of anti-VEGF antibody, both in control myoblast and miR126-5p transfected myoblast conditioned medium. Next, the level of VEGF protein was analyzed in medium conditioned by control or miR126-5p transfected myoblast (Fig. 8D). The increased level of VEGF was detectable in medium conditioned by myoblasts comparing to complete medium (Fig. 8D). However, we were not able to detect difference in VEGF protein level between control myoblast conditioned medium and miR126-5p transfection did not impact VEGF secretion by myoblast. Therefore, we concluded that miR-126-5p, up-regulated in response to SDF-1 treatment, changed the secretome of myoblasts and what impacted endothelial cell function. However, apart of VEGF additional molecules could be engaged in this effect.

## Discussion

The SC niche determines SC behavior and function^24^. The SC microenvironment is formed by extracellular matrix and different cells, including endothelial cells which reside in skeletal muscle and deliver complex signals to SCs. These signals change in the case of muscle injury and regeneration. Crosstalk between the vascular niche and muscle SCs plays a crucial role for skeletal muscle homeostasis and proper regeneration process^3, 25^. For example increasing vascular density improved the number of SCs in *mdx* mice, which are the mouse model of Duchenne muscular dystrophy^26^. Generally, in muscles SCs are located near or in direct contact with blood vessels, i.e., close to endothelium^25^. The key role in maintaining SC quiescence plays, *inter alia*, NOTCH signaling^27, 28^. The NOTCH ligands are expressed by muscle fibers, as well as by endothelial cells^28, 29^. The endothelial cell express DLL4 which is a ligand for the NOTCH receptor present on SCs, and the DLL4-NOTCH pathway promotes SC quiescence and self-renewal^25^. The interactions between SCs and endothelial cells are also regulated by VEGFA^25^. Both, quiescent and activated SCs express VEGFA that recruits endothelial cells^25^. The SC-derived myoblast migration is regulated by SDF-1^30^, the crucial regulator of cell mobilization and also angiogenesis^6, 8, 9, 31, 32^. However, little is known about the role of microRNA in SC niche homeostasis and changes during muscle regeneration, i.e., muscle fiber and also vasculature reconstruction. In the present study, we documented that SDF-1 treatment of SC-derived myoblasts increased the level such miRNAs as miR-126-3p and miR-339-5p-5p. On the other hand, SDF-1 treatment of cells in which *Cxcr4* or *Cxcr7* were down-regulated led to miR-126-3p, miR-324-3p, and miR-339-5p-5p down-regulation. The impact of SDF-1 on miR-126-3p and miR-126-5p expression was also observed in HUVECs^13, 33^. Importantly, *Cxcl12* mRNA encoding SDF-1 and *Cxcr4* mRNA are also predicted targets of miR-126a. In our study we showed that in mouse SC-derived myoblasts only miR-126-3p down-regulated the expression of *Cxcl12* encoding SDF-1 and *Ackr3* encoding CXCR7*.* Therefore, we conclude that miR-126-3p is an important regulator of SC-derived myoblast response to SDF-1. Moreover, we also showed that miR-126-3p inhibited SC-derived myoblast proliferation, migration, and fusion. These changes correspond to differences in the expression of proliferation marker *Ki67,* as well as *Pax7, Myod, Myf5, Myog* that regulate myogenic differentiation, and *Adam9, Vcam1* involved in myoblast fusion. Therefore, miR-126-3p is also a regulator of myogenic cell proliferation, migration, and differentiation.

Next, we showed that miR-126-5p increased *Vefga* level at mRNA level. Furthermore, the treatment of PMECs with medium conditioned by miR-126-5p transfected myoblasts promoted tubulogenesis. Importantly, blocking of VEGF under such conditions with anti-VEGF antibody impaired PMEC tubulogenesis. Moreover, miR-126-3p mimic decreased the level of *Vegfa* expression and medium conditioned by miR-126-3p transfected inhibited endothelial cell tubulogenesis. Therefore, we concluded that miR-126-5p played a crucial role in the regulation of the *Vegfa* level produced by SC-derived myoblasts exposed to SDF-1 and could also affect endothelial cell recruitment. Increased angiogenesis in critical limb ischemia in diabetic mouse was observed after transplantation of adipose tissue mesenchymal stromal cells overexpressing miR-126^34^. In such muscles, the *Vegf* level was higher than in control ones^34^. The regulation of *Vegf* expression via miR-126-3p depends on SPRED-1, which is the direct target of miR-126-3p^33, 35^. In our experiment we did not observe a decrease in SPRED-1 in response to miR-126-3p mimic transfection, but a significant increase in the level of mRNA encoding this factor in the response to miR-126-3p inhibitor . MiR-126 was shown to directly repress SPRED-1 and phosphoinositol-3 kinase regulatory subunit2 (PIK3R2/p85-β) that are negative regulators of the VEGF pathway in endothelial cells derived from mouse embryonic stem cells^35^. This mechanism was also described in other cell types, such as retinal neural stem cells derived from induced pluripotent stem cell-derived retinal neural stem cells^36^ and breast cancer cells^37^. MiR-126-3p and SDF-1 have also been documented to increase the migration and angiogenesis of HUVECs^33^. Exosomes obtained from miR-126 overexpressing mesenchymal stromal cells were shown to promote proliferation, migration, tubulogenesis, and to reduce endothelial cell apoptosis^38, 39^. Endothelial cells treated with such exosomes increased the levels of VEGF, EGF, PDGF, bFGF^38, 39^. Thus, exosomes were suggested to deliver miR-126 to endothelial cells^38, 39^. Furthermore, such exosomes increased the number of newly formed capillaries and accelerated the healing of wounds on the skin wound healing in vivo^39^.

## Conclusions

We concluded that miRNA-126a-3p and -5p play an important role in mouse SC-derived myoblast function and communication with endothelial cells. miR-126-3p down-regulated the level of transcripts encoding SDF-1 and its receptor and inhibited the migration and fusion of SC-derived myoblasts. The influence of miRNA-126-3p on myogenic differentiation was manifested by a decreased in the expression of numerous factors involved in myogenesis, such as *Pax7, Myod, Myf5, Myog, Adam9, Vcam1, and Myh3*. Importantly, miR-126-5p increased *Vefga* expression level. The conditioned medium from of miR-126-5p transfected SC-derived myoblasts promoted tubulogenesis of muscle endothelial cells. Therefore, we conclude that miR-126-5p upregulation effect SC-derived myoblasts secretome what could impact endothelial cell recruitment and differentiation. However, apart of VEGF additional molecules could be engaged in this effect.

## Methods

All experiments were performed in accordance with relevant guidelines and regulations. All methods are reported in accordance with ARRIVE guideline^40^. All studies fulfilled all obligatory requirements of National Ethics Committee for Animal Experiments, no experiments on living animals were performed.

### Satellite cell isolation and culture

Satellite cells (SCs) were isolated from Gastrocnemius, EDL, and Soleus muscles of 2–3-month-old male C57/BL6 according to method described by Rosenblatt and coworkers^35^. Next, the SCs were placed directly in culture plates or cover slides in culture plates. In each case the culture surface was coated with Matrigel Growth Factor Reduced (GFR) basement membrane matrix (Corning). Cells were expanded in DMEM with glucose 1 g/l supplemented with 10% HS, 20% fetal bovine serum (FBS, ThermoFisher Scientific) 1% penicillin/streptomycin, and 0.5% CEE and cultured under standard conditions, that is, 37 °C, 5% CO2. The medium was replaced every 2 days. Under such conditions, SCs started myogenic differentiation and formed SC-derived myoblasts.

### SC-derived myoblast transfection, SDF-1 treatment, and silencing of CXCR4 or CXCR7

One hour before transfection, the medium was changed to OptiMem (ThermoFisher Scientific) supplemented with 10% FBS. SC-derived myoblasts were transfected with Silencer Select predesigned siRNA (Life Technologies) complementary to Cxcr4 encoding mRNA (ID: s64091). Cxcr7 (ID: s64124). The siRNA duplexes were diluted in DMEM to a concentration of 100 pmol, and lipofectamine RNAiMAX (Life Technologies) was added according to the manufacturer's instructions. Mouse recombinant SDF-1 (10 ng/µl) was added to cell culture 24 h after transfection. Next, 48 h after SDF-1 treatment cells were collected and processed for RNA isolation and RNA sequencing.

SC-derived myoblasts were transfected using Lipofectamine 3000 (ThermoFisher Scientific) and 50 nM mirVana miRNA mimics or miRNA mimics and inhibitors: miR-126-3p, miR-126-5p, miR-324-3p, miR-339-5p (#12841, #10401, #12288, #12347, Ambion), according to the manufacturer's protocol. Control cells were transfected with the negative control #1 mimic (4464058, Ambion). The transfection procedure lasted for 48 h and then cells were washed with PBS and collected or further culture in medium that supports myogenic differentiation, that is, DMEM supplemented with 2% horse serum (HS; ThermoFisher Scientific) for 5 days. The cells were then collected for further analysis.

### Endothelial cell culture, treatment, and evaluation of tubulogenesis

SC-derived myoblasts were transfected with selected miRNAs or inhibitors as described above. After 48 h of transfection, the culture medium was changed to Complete Human Endothelial Cell Medium (CHECM, H-1168, Cell Biologics) and the cells were cultured for 24 h. Primary human skeletal muscle microvascular endothelial cells (PMECs; H-6220, CellBiologics) were seeded in culture plates covered with a thick layer of Matrigel GFR Basement Membrane Matrix in the density of 35,000 cells/cm^2^. The PMECs were cultured for 18 h either in Complete Human Endothelial Cell Medium or Complete Human Endothelial Cell Medium collected from control or transfected SC-derived myoblast cultures (SC-derived myoblasts conditioned medium). After 18 h of culture, the PMECs were fixed with 3% PFA. The number of branching points (nods) and the number of tubes formed in culture were calculated from at least 10 random fields of view for each variant of the experiment. Moreover, to evaluate the role of VEGF in tubulogenesis PMEC were cultured either in Complete Human Endothelial Cell Medium (CHECM) or Complete Human Endothelial Cell Medium collected from control or transfected SC-derived myoblast cultures (SC-derived myoblasts conditioned medium) in presence of 20 μg/ml anti-VEGF antibody (SP07-01, Invitrogen) for 16 h and fixed with 3% PFA.

### Proliferation assay

SC-derived myoblasts control or transfected with mimic miRNAs, were incubated in 0.5 µM carboxyfluorescein succinimidyl ester (CFSE, ThermoFisher) in PBS at 37 °C for 10 min. Cells were rinsed in PBS and cultured for 24 h or 48 h in growth medium under standard conditions. The cells were then rinsed in PBS, trypsinized, and subjected to flow cytometry analysis (CytoFLEX, Beckman Coulter) using CytExpert software. Unlabeled cells (negative control) and cells analyzed immediately after CFSE labeling (positive control) were included in each experiment. Three independent experiments were performed.

### Migration assay—scratch assay

Migration control or mimic miRNA transfected SC-derived myoblasts was analyzed using a scratch wound healing assay. Cells were scratched off the plate using a plastic tip to create the 'wound'. The wound healing manifested by the ability of cells to fill the created gap was observed. Pictures of the 'wound' area were taken at three time points: 0 h, 6 h, and after 24 h. The scratch area was calculated using Fiji and GraphPad 9 software (Prism, www.graphpad.com ). Three independent experiments were performed.

### Fusion index

SC-derived myoblasts were cultured and transfected as described above. After 48 h of transfection, cells were cultured in medium that supports myogenic differentiation for another 5 days. Finally, cells were fixed with 3% PFA for further analysis and stained with Giemsa. The fusion index was calculated as a percentage of cell nuclei present within myotubes compared to all visible nuclei. Three independent experiments were performed; nuclei were counted from 10 random fields of view.

### MicroRNA sequencing

RNA was isolated from SC-derived myoblasts transfected with siRNA complementary to mRNA encoding CXCR4 or treated with SDF-1. A total of three independent samples were collected. RNA was extracted in AROS Applied Biotechnology A/S Science Park Skejby (Denmark) with the QIAsymphony RNA extraction kit from QIAGEN (Germany) using the QIAsymphony SP Biorobot from QIAGEN (miRNA CT 400 program). MiRNA was quantified using a Qubit fluorometer and approximately 150 bp-long barcode libraries were created. The quality of the libraries was validated using the Bioanalyzer 2100 DNA High Sensitivity Kit (Agilent, USA). Libraries' concentrations were measured with the KAPA Library Quantification Kit during qPCR. Libraries were pooled with 30% spike in control libraries PhiX (Illumina) and hybridized to a sequencing flow cell. Single read 50 bp sequencing was performed using an Illumina HiSeq sequencer with the use of a TruSeq rapid SBS kit (Illumina, USA). The quality assessment of the raw reads was performed with the FastQC tool. First, only reads containing adapters were selected for further analysis. Later, adapters and low-quality reads were discarded. Then, reads with identical sequences were grouped/collapsed into one entry, and consequently were converted into read-count format. The miRNA expression profile was performed with the use of Partek Flow v6.0 and Partek Genomics Suite v6.6 software (Partek Inc., St. Louis, MO, USA). Briefly, reads were aligned with the mouse reference genome (assembly mm10) using Bowtie1. Next, miRBase (release 21) was used to provide annotations for RNA fragments assigned to the mouse genome. Finally, TMM normalized mapped reads were used to infer lists of differentially expressed miRNAs between analyzed groups. Unsupervised hierarchical clustering was performed with the use of the Partek Genomics Suite on normalized reads to visualize differentially expressed miRNAs. The p-value cut-off point for differentially expressed miRNAs between groups was established at the *p* value ≤ 0.05.

### Quantified real-time PCR and miRNA expression assay (qRT-PCR)

Total RNA, including miRNA fraction, was extracted from the control and mimic miRNA transfected SC-derived myoblasts s using the RNAqueous-Micro Total Isolation Kit (ThermoFisher Scientific), according to the manufacturer's protocol. RNA-based cDNA was synthesized using the RevertAid First-Strand cDNA synthesizer kit (ThermoFisher Scientific), according to the manufacturer's protocol, under the following conditions: 25 °C for 5 min, 42 °C for 90 min, and 70 °C for 5 min. mRNA levels were evaluated using quantitative real-time PCR analysis (qRT-PCR) with TaqMan assays (ThermoFisher Scientific) for the following genes: *Cxcl12* (Mm00445553_m1)*, Cxcr4* (Mm01996749_s1)*, Ackr3* (Mm04931206_s1)*, Ki67* (Mm01278617_m1)*, Cdc4* (Mm00504452_m1)*, Ptk2* (Mm00433209_m1)*, Rac1* (Mm01201653_mH)*, Acta1* (Mm00808218_g1)*, Pax7* (Mm01354484_m1)*, Myf5* (Mm00435125_m1)*, Myod* (Mm00521984_m1)*, Myog* (Mm00446194_m1)*, Myh3* (Mm01332463_m1)*, Camk2a* (Mm00437967_m1)*, Camsap1* (Mm01328273_g1)*, Camta1* (Mm01051596_m1)*, Camta2* (Mm00626346_m1)*, Cask* (Mm00438021_m1)*, Fst* (Mm00514982_m1)*, Spred1* (Mm01277511_m1)*, Spry1* (Mm01285700_m1)*, Spry2* (Mm00442344_m1)*, Vegfa* (Mm00437306_m1)*, Vcam1* (Mm01320970_m1)*, Adam9* (Mm01218460_m1). The expression of hypoxanthine phosphoribosyltransferase 1 (*Hprt1*; Mm03024075_m1) was used as a reference gene expression for further calculations. The reaction was carried out with TaqMan Gene Expression Master Mix (ThermoFisher Scientific) using LightCycler96 (Roche) under the following conditions: preincubation 2 min, 50 °C; preincubation 10 min, 95 °C; amplification 40 cycles 15 s, 95 °C and 1 min, 60 °C. All reactions were performed in duplicates. Expression levels were calculated using 2-(ΔCt) formula based on the relative expression of Hprt1. MiRNA reverse transcription was performed with the TaqMan MiRNA reverse transcription kit (ThermoFisher Scientific) and TaqMan miRNA assays (ThermoFisher Scientific) under the following conditions: 30 min, 16 °C; 30 min 42 °C; 5 min, 85 °C. MiRNA levels were assessed using quantitative real-time PCR (qPCR) analysis. RT primers and TaqMan probes were used for specific miRNA: miR-126-3p (#002228), miR-126-5p (#000451), miR-324-3p (02509), miR-339-5p (#002257), U6 (#001973). The average expression of non-coding U6 snRNA was used as a reference for further calculations. The reaction was carried out with TaqMan Universal Master Mix II, without UNG (ThermoFisher Scientific) using LightCycler96 (Roche) under the following conditions: preincubation 2 min, 50 °C; preincubation 10 min, 95 °C; amplification (40 cycles) 15 s, 95 °C and 1 min, 60 °C. All reactions were performed in duplicates. Three independent experiments were performed.

### ELISA

The complete OptiMem or CHECM, or complete OptiMem collected from cultures of control or transfected SC-derived myoblasts (48 h after transfection) or CHECM conditioned with control or transfected SC-derived myoblasts presence for 24 h, was analyzed using Human VEGF SimpleStep ELISA kit (ab222510, Abcam), accordingly to manufacture’s instruction. 562 nm absorbance was measured using a microplate reader BioTek ELx800 (Agilent) with Gen5 software (Agilent). Three independent experiments were performed in duplicates. The average results for each experiment were shown on graphs. The graphs were performed using GraphPad 9 software (Prism, www.graphpad.com ).

### Statistical analysis

The Gaussian distribution of values was analyzed with the Shapiro–Wilk normality test. The fold change was calculated by comparing the average values of the non-treated samples to those of all samples. Data were analyzed using the one-way ANOVA test and post hoc with Dunnett’s multiple comparisons test. All data was compared to results obtained from analyzes of the control group, that is, non-treated cells. Differences were considered statistically significant when *p <* 0.05 (marked with asterisks, *p < 0.05; **p < 0.005; ***p < 0.001; ****p < 0.0001). The mean value and standard deviation were shown in each graph presented. All statistical analyzes and graphs were performed using GraphPad 9 software (Prism, www.graphpad.com ).

## Data Availability

The data sets generated and analyzed during the current study are available on request from the corresponding author. Department of Cytology, Faculty of Biology, University of Warsaw, Miecznikowa 1 St, 02-096 Warsaw, Poland. ArrayExpress accession number E-MTAB-12810.
